# Optimising Neurosonography in the NICU: An Audit of 130 Cases and Proposal of a Referral and Reporting Protocol

**DOI:** 10.7759/cureus.110309

**Published:** 2026-06-05

**Authors:** Khushboo Jain, Ashish Kaushik, Tamanna Jindal, Dhananjay Patil, Divya Pandey, Sachin Gupta

**Affiliations:** 1 Medical School, Lady Hardinge Medical College, Delhi, IND; 2 Radiodiagnosis and Interventional Radiology, Frank Institute of Medical Sciences, Sonipat, IND; 3 Radiodiagnosis Breast Imaging, All India Institute of Medical Sciences, Rishikesh, Rishikesh, IND

**Keywords:** cranial ultrasound, neurosonography, nicu care, referral criteria for neurosonography, reporting template for neurosonography

## Abstract

Introduction

Neurosonography is a first-line, safe bedside modality for evaluating neonates at risk of intracranial pathology. International guidelines recommend routine screening in every preterm infant and targeted scans in neonates with systemic risk factors. However, in practice, it is frequently both underutilised (missed scans in at-risk neonates) and overutilised (repeated scans in low-risk infants without clinical indication). This results in either delayed detection of critical conditions or unnecessary imaging with minimal clinical yield. We conducted an audit of 130 neonatal neurosonograms at our tertiary care centre to evaluate current utilisation patterns, assess clinical yield, and analyse the impact on management. Based on the findings, we propose a referral and reporting protocol as an exploratory instrument to improve neonatal outcomes. However, this hypothesis needs to be validated in further prospective studies.

Methods

We retrospectively analysed 130 consecutive neurosonograms performed over 12 months in neonates admitted to the neonatal intensive care unit (NICU) of Frank Institute of Medical Sciences, India. Data collected included clinical indication for scan, birth history, neurosonographic diagnosis, and outcome. Based on the current American Academy of Pediatrics (AAP)/American Institute of Ultrasound in Medicine (AIUM) guidelines and clinical judgement, referrals were classified as: Appropriate, possibly appropriate, inappropriate and indeterminate. To assess underutilization, delivery records during the same period were reviewed. Infants were also stratified into high-risk and low-risk groups based on international criteria. The proportion of high-risk neonates who did not undergo neurosonography was calculated. Statistical analysis compared the yield and management impact between appropriate and inappropriate referrals using chi-square analysis.

Results

Germinal matrix haemorrhage (GMH) was detected in 18 cases (13.8%). Periventricular leukomalacia (PVL) was identified in 19 cases (14.6%). Other findings included hydrocephalus or VP shunt-related changes in five cases (3.8%), cephalohematoma or caput succedaneum in five cases (3.8%), meningitis in one case (0.7%), and cerebral abscess in one case (0.7%). The majority of scans were normal. Diagnostic yield was significantly higher in appropriate referrals compared to the inappropriate group (chi-square p = 0.038). Twenty-four scans (18%) directly influenced clinical management, representing significant clinical implications. Of 24 neonates meeting the high-risk criteria, nine (36%) did not receive neurosonography, suggesting underutilisation.

Discussion

Our audit shows a wide variation in neurosonography utilisation, with underuse in high-risk neonates and overuse where the yield is low. The diagnostic yield was significantly higher in appropriate referrals, suggesting an association between referral quality and imaging value. We have developed a referral checklist and reporting template. The checklist has been developed to be used at the point of clinical decision-making. The reporting template includes disease-specific action triggers to maximise clinical effect. However, both tools are proposed as exploratory instruments derived from a single-centre retrospective audit.

Conclusion

Neurosonography is an essential tool for NICU care. Its clinical impact may improve when guided by structured referral protocols and standardised action-oriented reporting.

## Introduction

Neurosonography, also known as cranial ultrasound, is a first-line, safe bedside modality for evaluating neonates at risk of intracranial pathology. International guidelines [1-4] recommend routine screening in every preterm infant and targeted scans in neonates with neurological or systemic risk factors. However, in practice, neurosonography is frequently both underutilised (missed scans in at-risk neonates) and overutilised (repeated scans in low-risk infants without clinical indication). This results in either delayed detection of conditions, such as germinal matrix haemorrhage (GMH) and periventricular leukomalacia (PVL), or unnecessary imaging with minimal clinical yield.

We conducted an audit of 130 neonatal neurosonograms at our tertiary care centre to evaluate current utilisation patterns, assess clinical yield, and analyse the impact on management. Based on the findings, we propose a referral and reporting protocol as an exploratory instrument for better neonatal outcomes. However, this hypothesis needs to be validated in further prospective studies.

## Materials and methods

Objective

We audited all neurosonograms at our centre over 12 months, assessing their appropriateness, diagnostic yield, and impact on management. We aim to classify indications for neurosonography and assess the appropriateness of referrals. We describe the spectrum of neurosonographic findings across 130 neonates. We also determine how frequently scan results altered management or influenced clinical outcomes. We aim to identify existing gaps in the referral and reporting process at our institution and to propose or refine a referral and reporting protocol that can be implemented even in low-resource centres. This will help us identify practice gaps, the correction of which might contribute to reducing neonatal morbidity and mortality in future prospective work.

Methods

We retrospectively analysed 130 consecutive neurosonograms performed over 12 months in neonates admitted to the neonatal intensive care unit (NICU) of Frank Institute of Medical Sciences, Sonipat, Haryana, India. For each case, data were collected across four key domains: the clinical indication for the scan; birth history encompassing gestational age, birth weight, term or preterm status, obstetric history, presence of birth asphyxia, and duration of NICU stay; the final neurosonographic diagnosis; and the subsequent clinical action or outcome, including any changes in surgical, medical, or monitoring management.

Referral appropriateness was evaluated against current American Academy of Pediatrics (AAP)/American Institute of Ultrasound in Medicine (AIUM) guidelines and clinical judgement, and each referral was classified into one of four categories. Referrals were considered appropriate when they fulfilled a clear, guideline-backed indication; possibly appropriate when the referral could be justified within the full clinical context but did not meet a strong guideline indication ('grey zone' referrals); inappropriate when no identifiable clinical indication was present, often done for parental reassurance; and indeterminate when sufficient clinical information could not be retrieved or no indication could be deciphered from available records.

To evaluate underutilisation, delivery records from the same 12-month period were reviewed. Infants were stratified into high-risk and low-risk groups based on international guidelines, including prematurity at or below 32 weeks of gestational age, very low birth weight, perinatal asphyxia, suspected sepsis or meningitis, and congenital anomalies. The proportion of high-risk neonates who did not undergo neurosonography was calculated. Statistical analysis comparing the yield and management impact of appropriate versus inappropriate referrals was performed using the chi-square test.

Professional practice standards in neonatal neurosonography

Technical performance standards established by the American College of Radiology (ACR), American Institute of Ultrasound in Medicine (AIUM) [1], Society for Pediatric Radiology (SPR), and Society of Radiologists in Ultrasound (SRU) provide structured guidance for optimal image acquisition and reporting of neonatal cranial ultrasound. These guidelines outline a systematic approach to image acquisition in coronal and sagittal images through the anterior fontanelle. Supplementary views through the mastoid fontanelle are also acquired for posterior fossa evaluation. High-frequency transducers (7.5-12 MHz) are recommended for best resolution, and examinations must adhere to ALARA (As Low As Reasonably Achievable) safety principles for safe evaluation [5,6]. 

Prerequisites

Neurosonography has a learning curve, and adequate training is required to ensure reliable and clinically meaningful results. It requires a combination of technique, knowledge, and skill to arrive at appropriate results. Diagnostic confidence among radiologists may be limited by inadequate training exposure and suboptimal technique. The acoustic windows available for imaging are determined largely by the patency of the fontanelles, which varies with postnatal age. The three most clinically useful windows are the anterior fontanelle, posterior fontanelle, and mastoid fontanelle.

**Figure 1:**
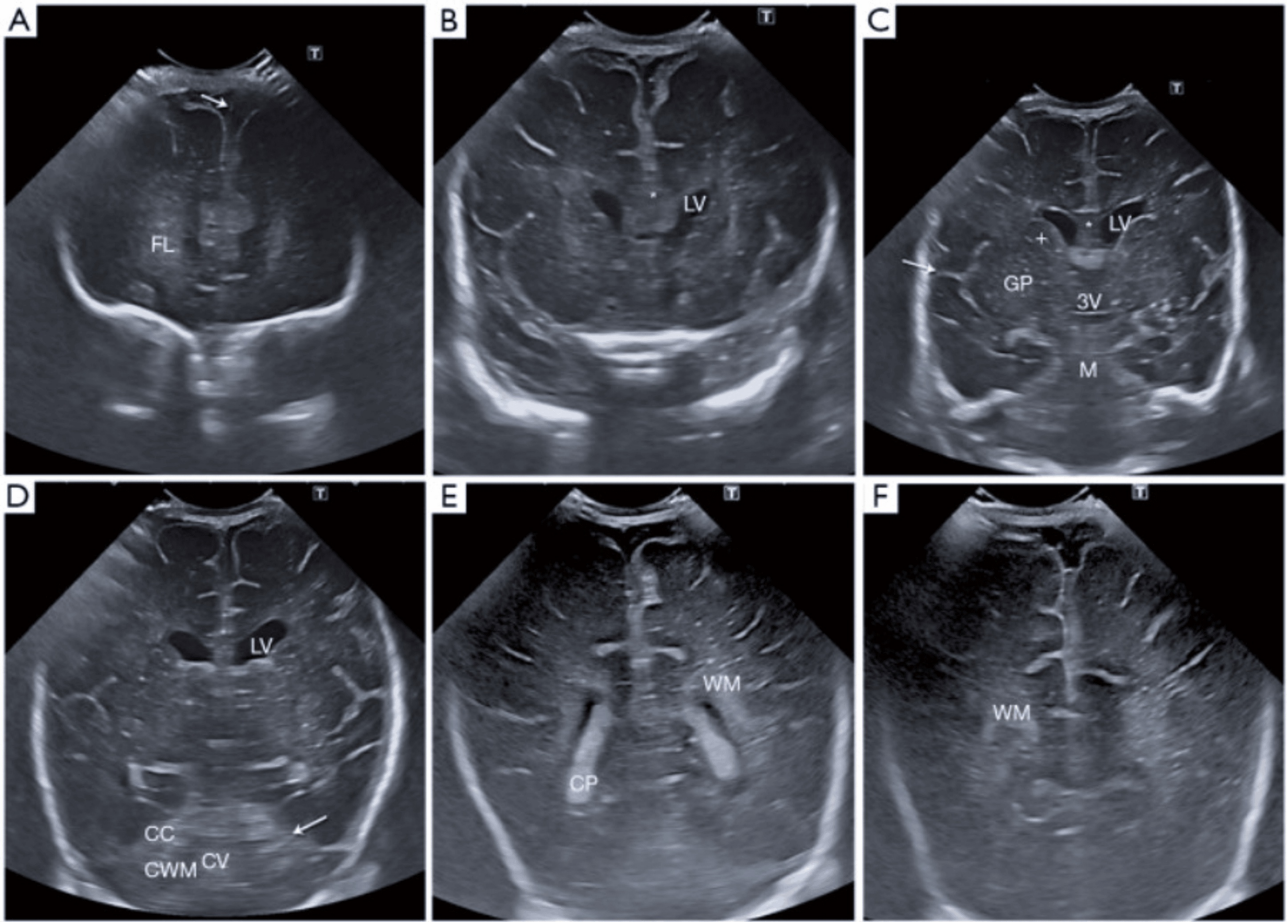
Cranial ultrasonography was performed on a 14-day-old female neonate with intrauterine growth restriction using an 11 MHz transducer. (A) This coronal section at the level of the frontal lobes shows the interhemispheric fissure. (B) The coronal plane through the frontal horns of the lateral ventricles reveals the corpus callosum. (C) This coronal image at the level of the third ventricle displays the lateral ventricles, basal ganglia structures, midbrain, corpus callosum, and Sylvian fissures. (D) A section at the cerebellar level illustrates the vermis, cerebellar hemispheres, cortex, and tentorium. (E) An image through the ventricular trigone shows the choroid plexus and nearby periatrial white matter. (F) The posterior coronal section at the level of the occipital lobes depicts the periventricular white matter. Reproduced from Caro-Domínguez et al. [1]: distributed under the Creative Commons Attribution-NonCommercial-NoDerivatives 4.0 International License (CC BY-NC-ND 4.0).

**Figure 2:**
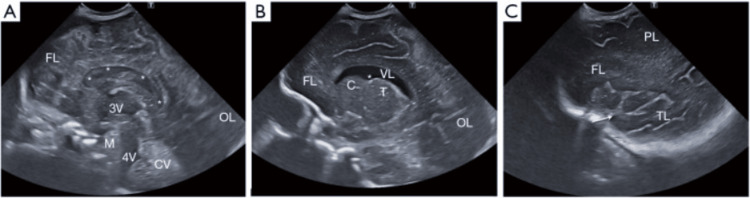
Sagittal and parasagittal cranial ultrasonographic views. (A) Midline sagittal image showing the corpus callosum, third and fourth ventricles, cerebral hemispheres from frontal to occipital regions, midbrain, and cerebellar vermis. (B) Parasagittal section illustrating the caudothalamic groove located between the caudate nucleus anteriorly and the thalamus posteriorly. (C) Lateral parasagittal view depicting the frontal, parietal, and temporal lobes with visualization of the Sylvian fissure. Reproduced from Caro-Domínguez P et al. [1]: distributed under the terms of the Creative Commons Attribution-NonCommercial-NoDerivatives 4.0 International License (CC BY-NC-ND 4.0).

**Figure 3:**
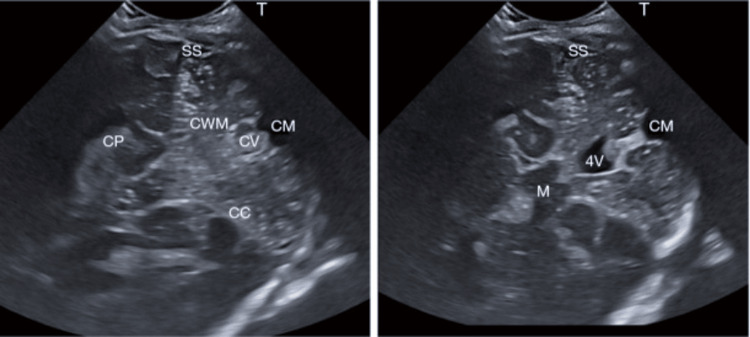
Coronal ultrasonographic images of the posterior fossa were obtained through the mastoid fontanelle in the same patient. The images show the cerebellar hemispheres, including cortical and white matter, cerebellar vermis, sigmoid sinus, fourth ventricle, and cisterna magna. Above the tentorium, the temporal lobe and temporal horn of the lateral ventricle are visible, with the choroid plexus identified within the ventricular cavity. Reproduced from Caro-Domínguez P et al.: distributed under the terms of the Creative Commons Attribution-NonCommercial-NoDerivatives 4.0 International License (CC BY-NC-ND 4.0).

Imaging is performed through three fontanelle windows, namely, the anterior, posterior, and mastoid. The anterior fontanelle is the primary access point. Six coronal and five sagittal planes are obtained through it, as shown in Figures 1-2. The coronal planes progress from the frontal lobes anteriorly to the occipital lobes posteriorly. The third coronal image is at the caudothalamic groove, which is particularly important for detecting germinal matrix haemorrhage, and the fifth for assessing periventricular white matter, the region most vulnerable to PVL. The sagittal planes include a midline view of the corpus callosum, brainstem, and cerebellar vermis. Two parasagittal views of the lateral ventricles, and two lateral views at the Sylvian fissures are also taken. Two additional coronal images through the mastoid fontanelle (Figure 3) offer superior posterior fossa visualisation, although they are technically more demanding to obtain [1].

## Results

Spectrum of findings

**Figure 4:**
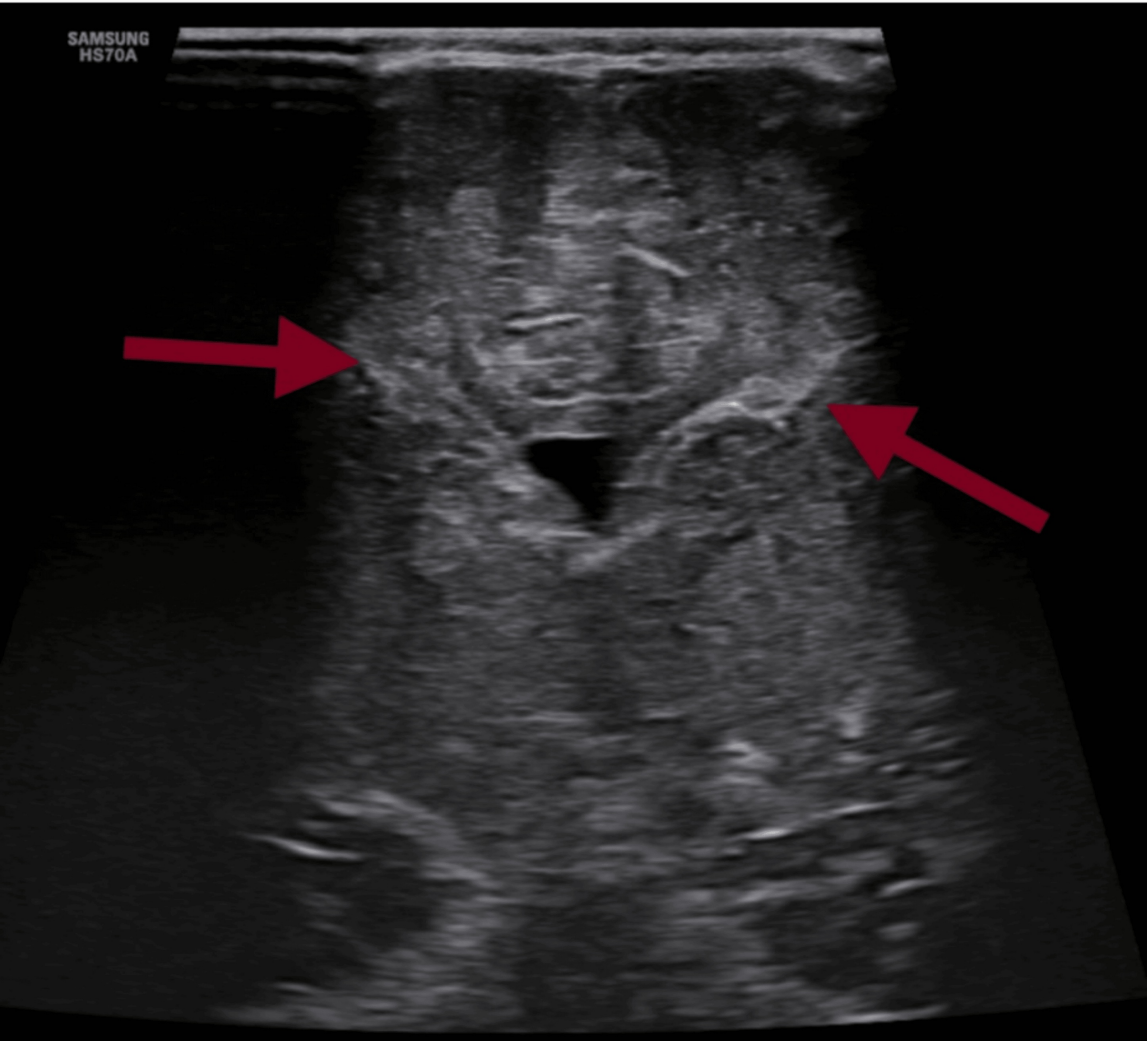
Coronal section of the brain showing increased periventricular echogenicity (marked by the arrows) seen in Periventricular leucomalacia (PVL) - Grade I.

**Figure 5:**
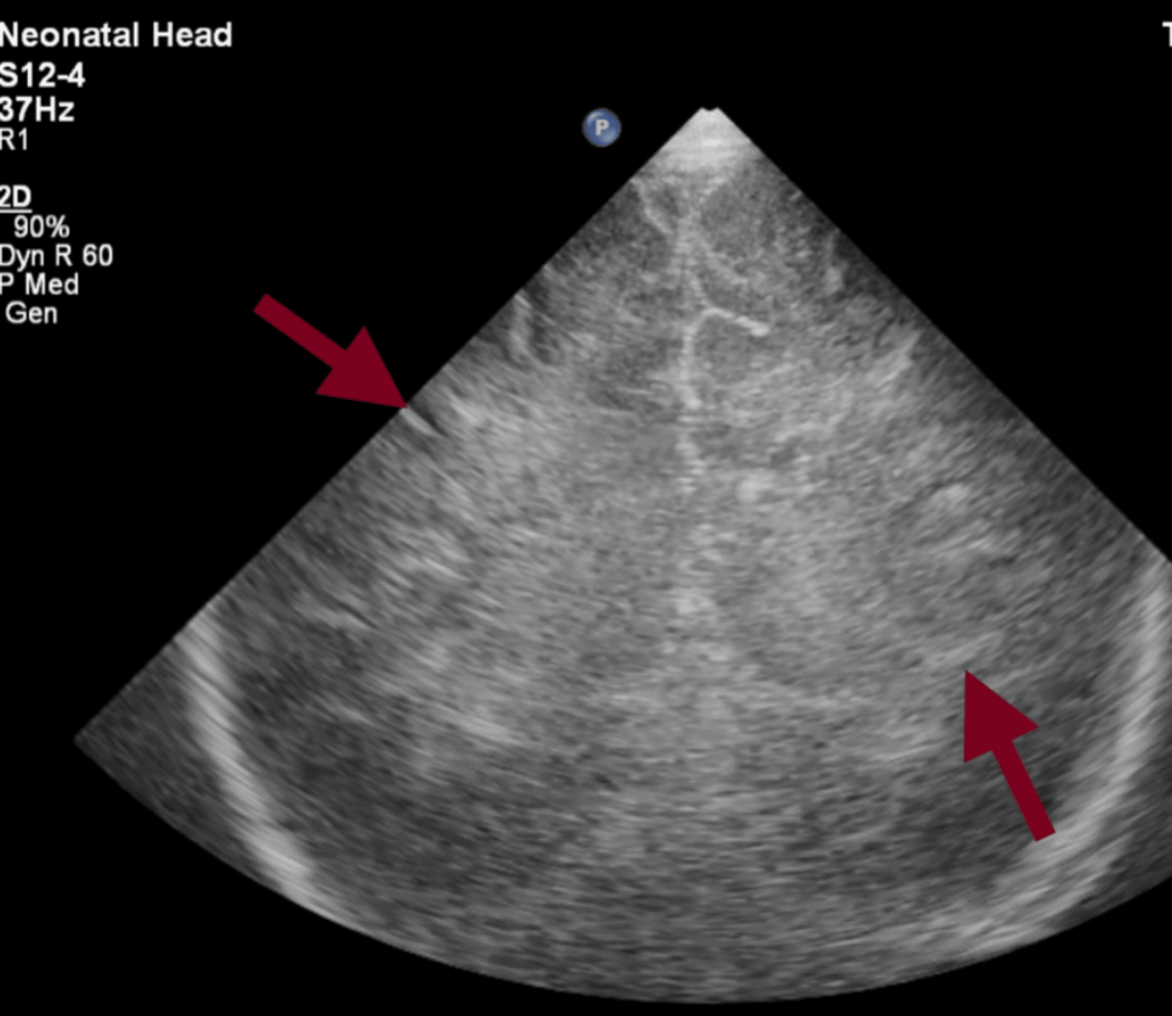
Coronal section of the brain showing increased sulcal echogenicity (marked by the arrows) seen in a suspected case of meningitis

**Figure 6:**
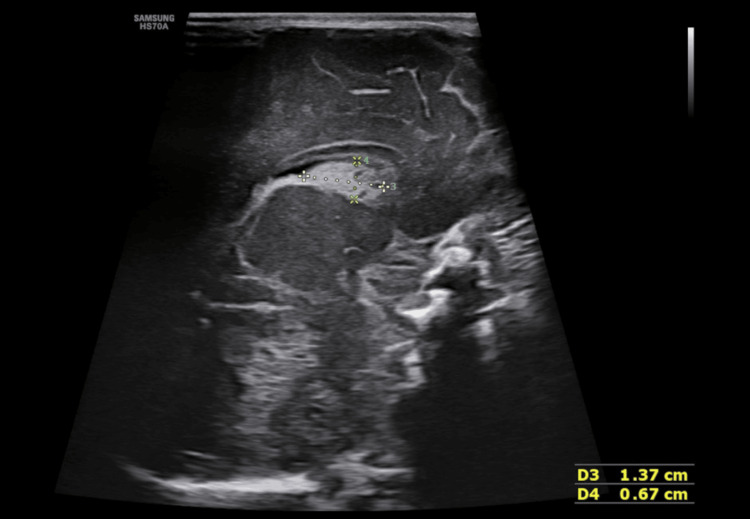
Sagittal section of the brain showing echogenicity in the caudo-thalamic groove with no intraventricular extension - Suggestive of Germinal matrix haemorrhage - I

**Figure 7:**
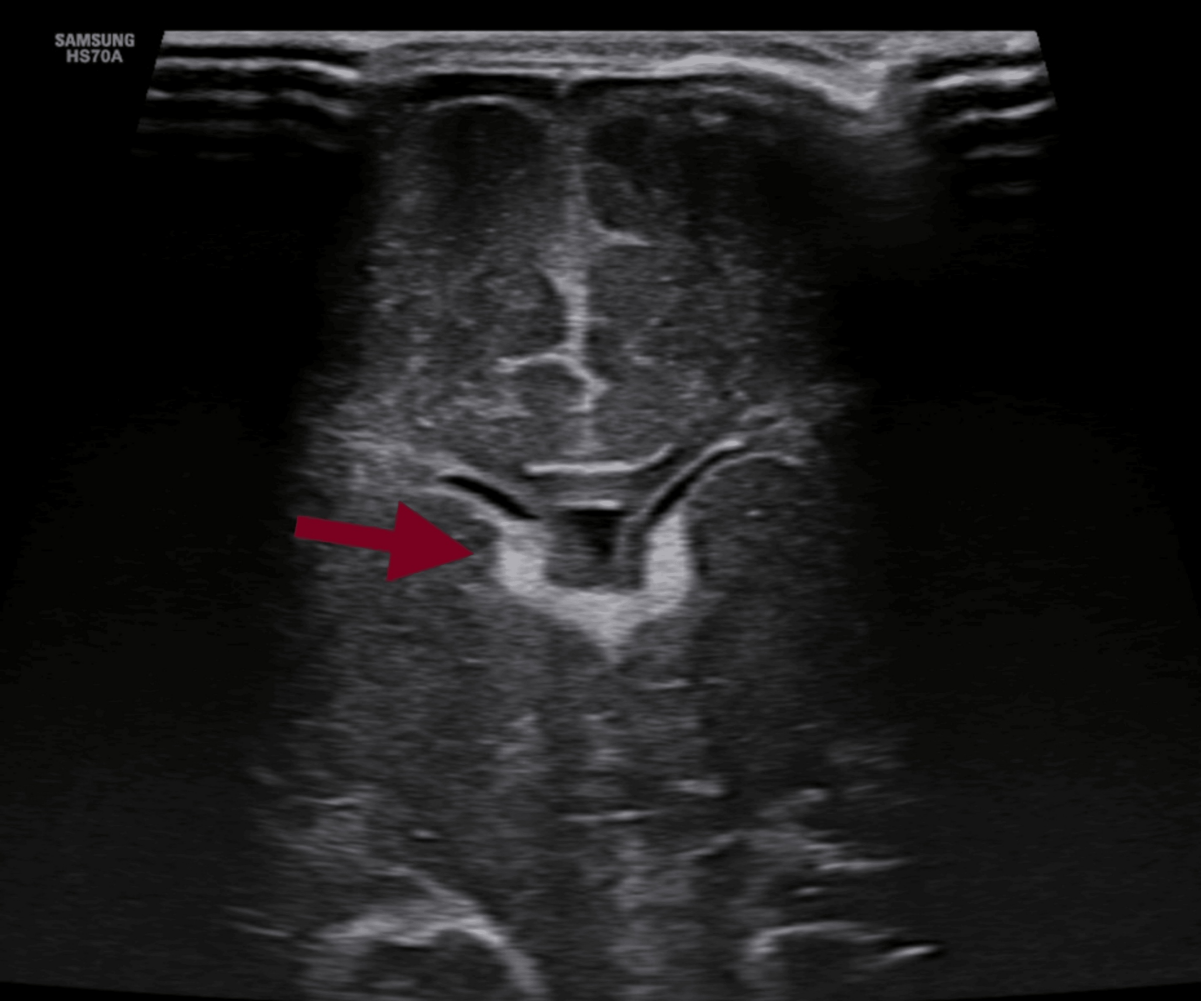
Coronal section of the same patient shows increased bilateral periventricular echogenicity confined to the bilateral germinal matrix - Suggestive of Germinal matrix haemorrhage (Grade I)

**Figure 8:**
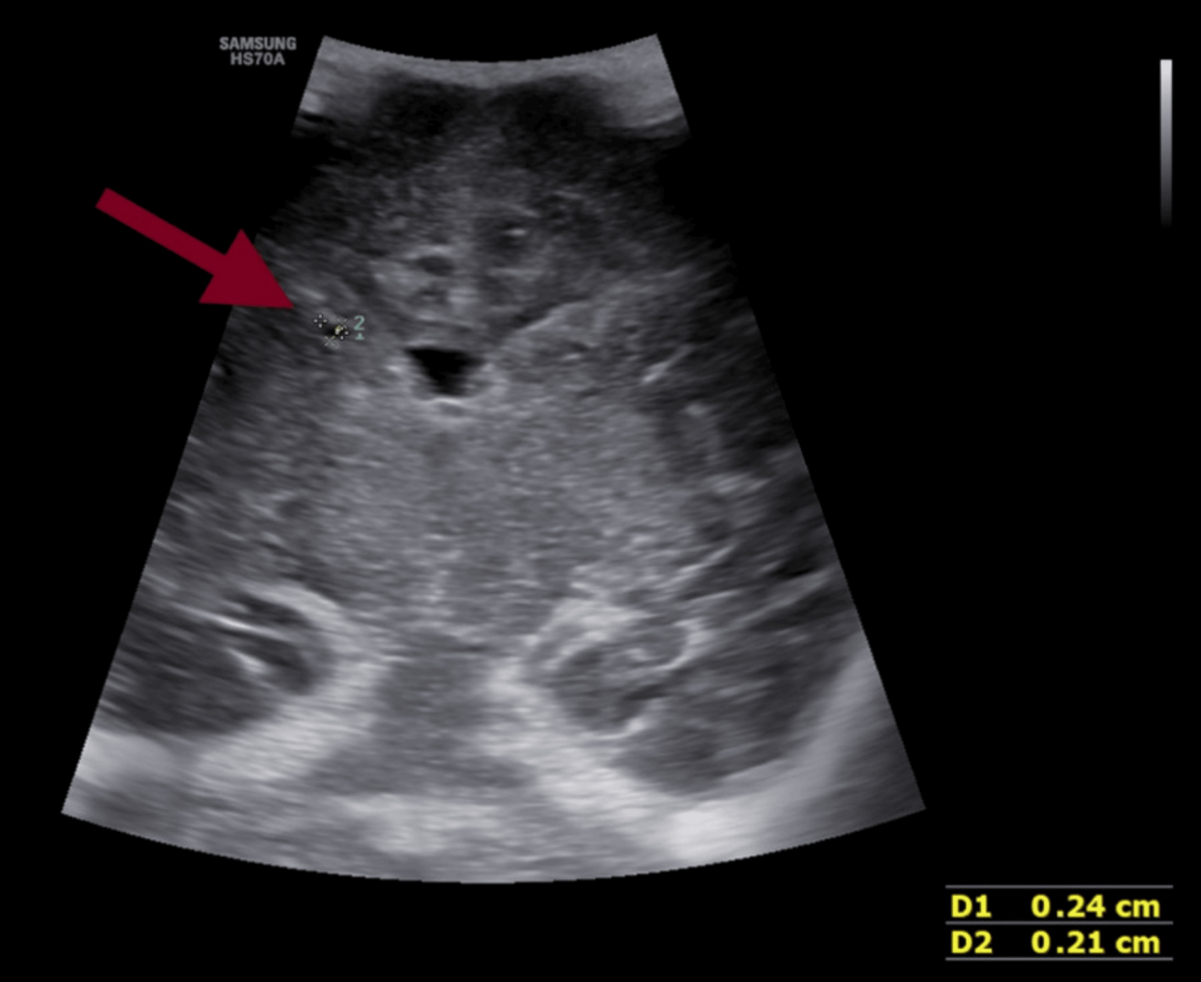
Coronal section of brain showing increased periventricular echogenicity with a small cyst (marked by arrow) in the right periventricular area, measuring ~ 2.4 x 2.1 mm - Suggestive of Periventricular leucomalacia (PVL) - Grade II.

**Figure 9:**
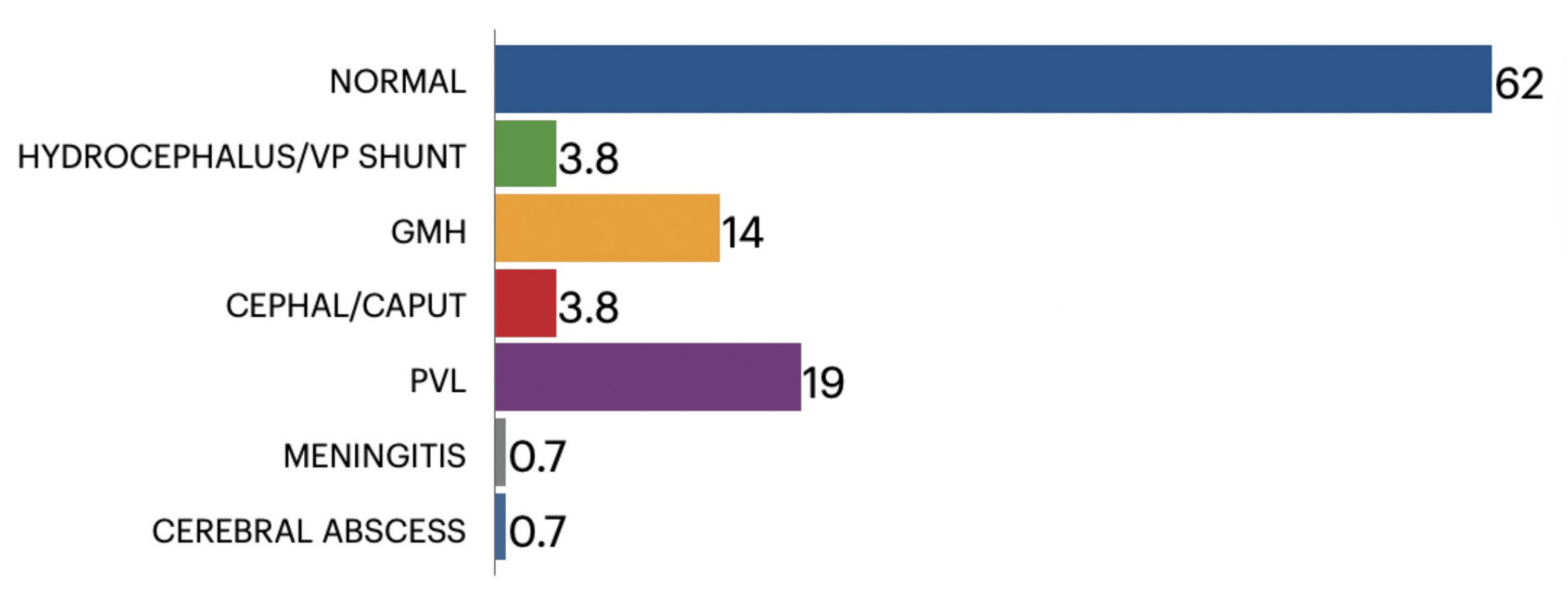
Spectrum and prevalence of cranial ultrasound findings in the study population, with normal scans comprising the majority and GMH-IVH and PVL being the most common abnormalities. GMH - Germinal matrix haemorrhage IVH - Intraventricular haemorrhage PVL - Periventricular leucomalacia

Neurosonography revealed a range of clinically significant findings across the 130 cases examined (Figures 4-8). Germinal matrix haemorrhage was detected in 18 neonates (13.8%), distributed across all four grades: grade I in 12 cases (9.2%), grade II in four cases (3%), and grades III and IV in one case each (0.7%). PVL was identified in 19 cases (14.6%), making it the most frequently detected abnormality overall. Hydrocephalus or ventriculoperitoneal (VP) shunt-related changes were present in five cases (3.8%), with each case representing a direct indication for neurosurgical consult. Meningitis or ventriculitis was detected in one case (0.7%), and a cerebral abscess was identified in one case as well (0.7%), both representing rare but life-threatening findings. By contrast, cephalohematoma or caput succedaneum, a benign extracranial finding, was noted in five cases (3.8%). The majority of examinations were reported as normal, reflecting the challenge of maintaining diagnostic yield in a population where referral appropriateness varies considerably, as shown in Figure 9. 

**Figure 10:**
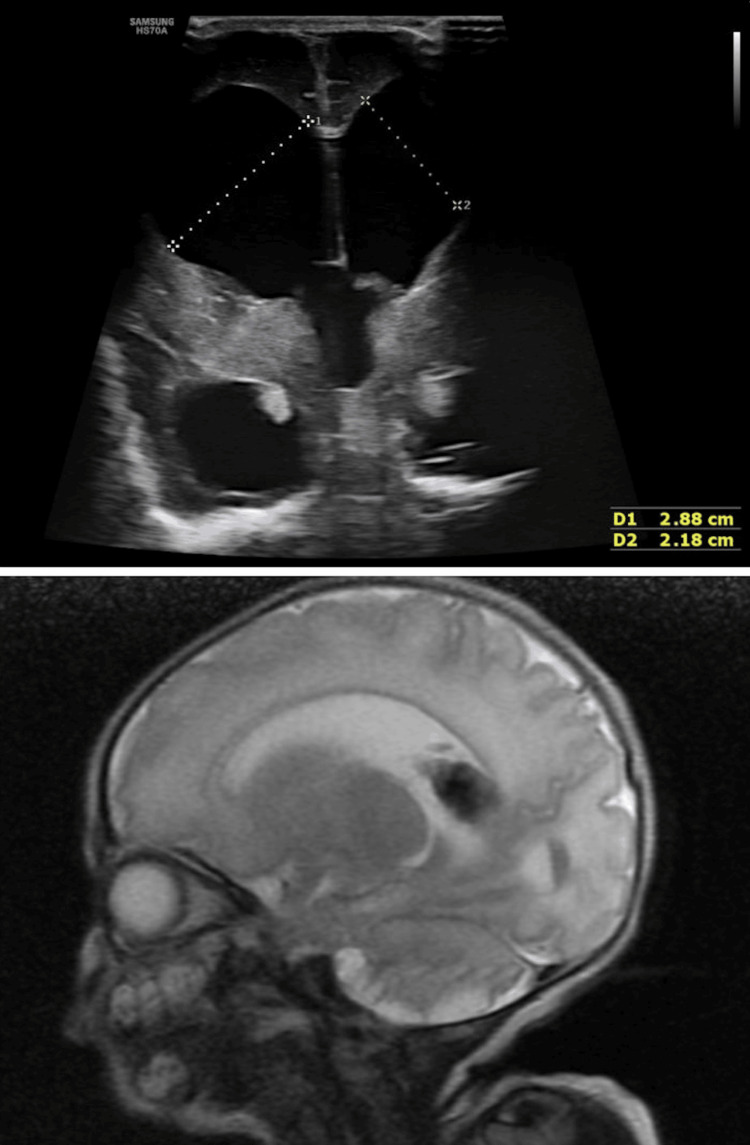
Neurosonography image on the top of a preterm baby shows coronal view with dilatation of bilateral lateral ventricles - suggestive of hydrocephalus. MRI image at the bottom of the same patient correlates with the neurosonography findings. The baby was then taken up for VP shunt insertion. Neurosonography, if done and interpreted properly,y has similar efficacy as MRI and can be used in low-resource settings when MRI is not always available or possible in sick/unstable patients. VP = ventriculo-peritoneal

MRI was performed in one infant (Figure 10) and demonstrated findings that were concordant with the neurosonography report in this individual case. This case highlights the potential utility of neurosonography as an initial bedside imaging modality when performed by a trained operator, particularly in resource-limited settings or where MRI is unavailable or not feasible. These include conditions requiring emergency neurosurgical interventions and serve as a viable alternative in resource-limited settings or in haemodynamically unstable neonates who cannot safely undergo MRI [5,6]. However, given that this represents a single observation, no generalisable conclusions regarding diagnostic equivalence, sensitivity, or specificity relative to MRI can be drawn. MRI remains the reference standard for comprehensive evaluation and should be performed whenever further characterisation of abnormalities is required.

GMH-IVH [7-9] coming out in 18 cases (14%) is still significant because these babies are at risk of progression, hydrocephalus, and neurodevelopmental delay. PVL [10,11] accounting for 19 cases (14.56%) with implications for long-term prognosis and early rehab planning. Hydrocephalus/VP shunt [12,13] was seen in five (3.8%) of the cohort and is a direct indication of neurosurgical management. Meningitis/ventriculitis/abscess was present in two (1.4%) cases. Although rare, these findings carry significant clinical consequences and underscore the importance of maintaining neurosonography availability for at-risk neonates.

Classification of referrals based on the AIUM and AAP guidelines

Case referrals were classified against established international guidelines from both the AIUM and the AAP. The 2020 AIUM consensus [2] gives a broad range of clinical scenarios warranting neurosonography in infants. It includes abnormal head circumference growth, haemorrhagic or parenchymal abnormalities in preterm and term infants, ventriculomegaly and hydrocephalus, vascular anomalies, suspected hypoxic-ischaemic injury, neonates receiving therapeutic hypothermia or extracorporeal membrane oxygenation, congenital brain malformations, neurological symptoms such as seizures or macrocephaly, confirmed or suspected intracranial infection, cranial trauma, craniosynostosis, surveillance of previously documented prenatal or postnatal abnormalities, and preoperative baseline assessment.

The AAP recommendations [3] give indications for the preterm population more specifically. Routine cranial ultrasound screening is advocated for all neonates born at or below 30 weeks of gestational age, as well as for selected older preterm infants presenting with additional risk factors for brain injury, including placental abruption, requirement for vigorous resuscitation, haemodynamic instability requiring vasopressors, significant acidosis, prolonged mechanical ventilation, confirmed sepsis, or pneumothorax. The AAP [3] recommends an initial scan between seven and 10 days of life for infants born at or below 30 weeks, with earlier imaging indicated if neurological signs suggest significant brain injury before this window. Follow-up scans are advised at four to six weeks of age, and again at term-equivalent age or prior to hospital discharge. Infants with abnormal initial findings should undergo serial imaging as clinically guided by both chronological and corrected gestational age. The guidelines specify that standard views should include the anterior and mastoid fontanelle windows, with posterior fontanelle and Doppler vascular imaging available as supplementary tools. Computed tomography is no longer recommended as part of routine preterm brain imaging. MRI is not indicated routinely for infants born below 30 weeks, with its optional use at term-equivalent age.

**Figure 11:**
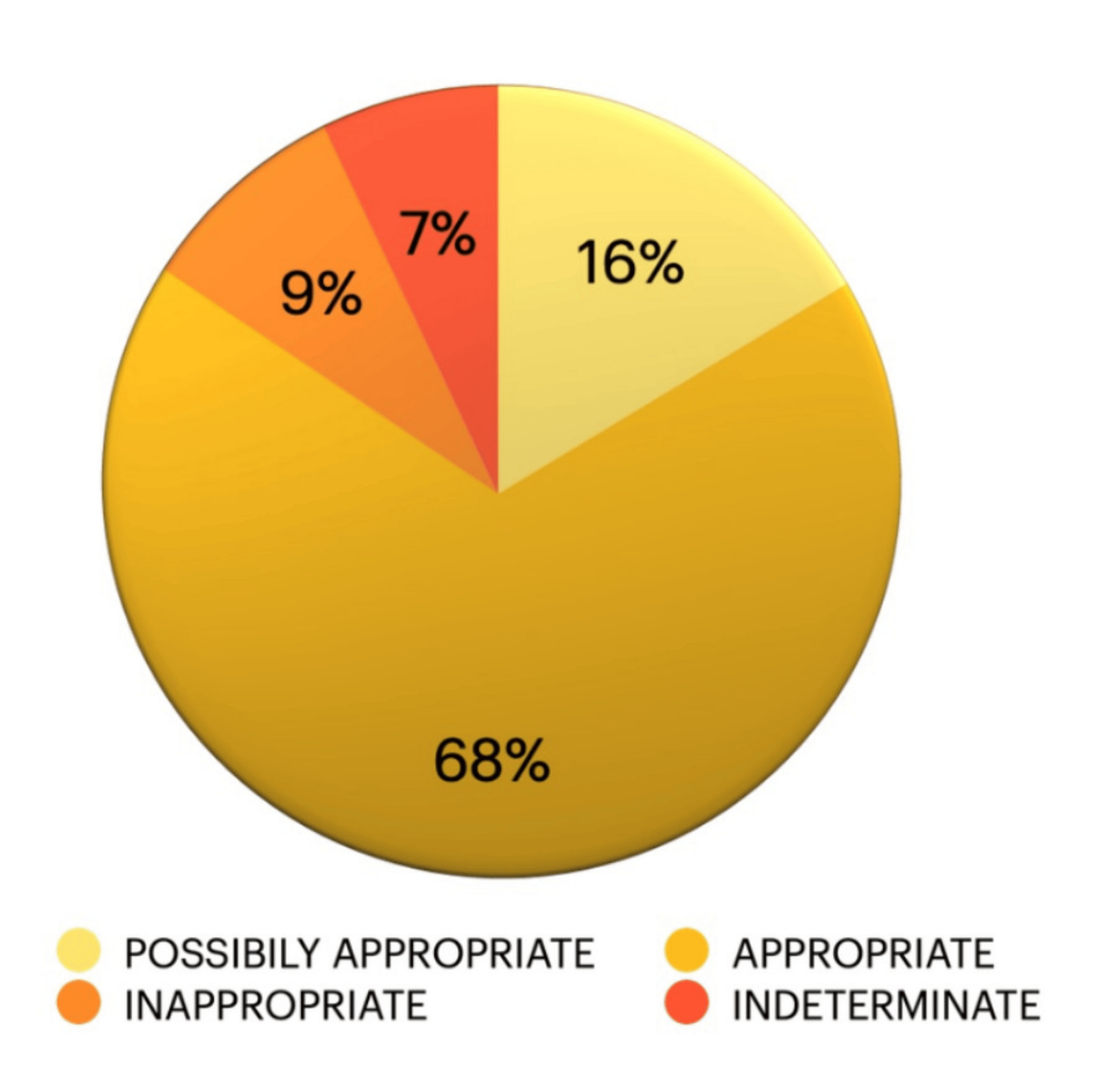
Distribution of imaging referrals categorised according to appropriateness criteria (American Academy of Pediatrics (AAP) and American Institute of Ultrasound in Medicine (AIUM) guidelines), showing the proportion of appropriate, possibly appropriate, inappropriate and indeterminate scans.

Considering these indications, the 130 referrals in our cohort were stratified as follows: 88 scans (68%) were classified as appropriate, representing clear guideline-backed indications; 21 scans (16%) were possibly appropriate, reflecting borderline clinical contexts; 12 scans (9%) were deemed inappropriate, with no identifiable indication, most commonly performed for parental reassurance; and 9 scans (7%) were indeterminate owing to incomplete or irrecoverable clinical data (Figure 11).

Diagnostic yield

Using the above data, the diagnostic yield or positive result/abnormal scan was calculated. The diagnostic yield was highest in appropriate referrals compared to inappropriate referrals. In our cohort, nearly two-thirds of neurosonograms were normal, showing frequent low-yield or inappropriate referrals. However, the abnormal findings represented conditions with major prognostic and management implications (GMH-IVH, PVL, hydrocephalus, and HIE). This highlights the dual challenge: to reduce unnecessary scans and ensure timely detection of high-impact pathologies. 

To assess the association between referral appropriateness and diagnostic yield, a chi-square test was performed, as shown in Table 1. The result was statistically significant (p = 0.038), suggesting that referral category and diagnostic yield are not independently distributed in this cohort. While this finding is consistent with the value of structured referral practice, it should be interpreted within the constraints of a single-centre retrospective audit with a small sample size.

**Table 1:** Chi-square analysis of referral appropriateness and imaging outcomes, demonstrating a statistically significant association between referrals and abnormal imaging findings. This highlights the need for a structured referral criterion. Chi square = 4.2583 The p value is 0.0391. Significant at p < 0.05.

	Normal	Abnormal
Appropriate referrals	43	45
Inappropriate referrals	9	2
Total	52	47
Expected frequencies		
Appropriate referrals	46.22	41.778
Inappropriate referrals	5.778	5.222
Chi-square points		
Appropriate referrals	0.225	0.249
Inappropriate referrals	1.797	1.988

Management impact

**Figure 12:**
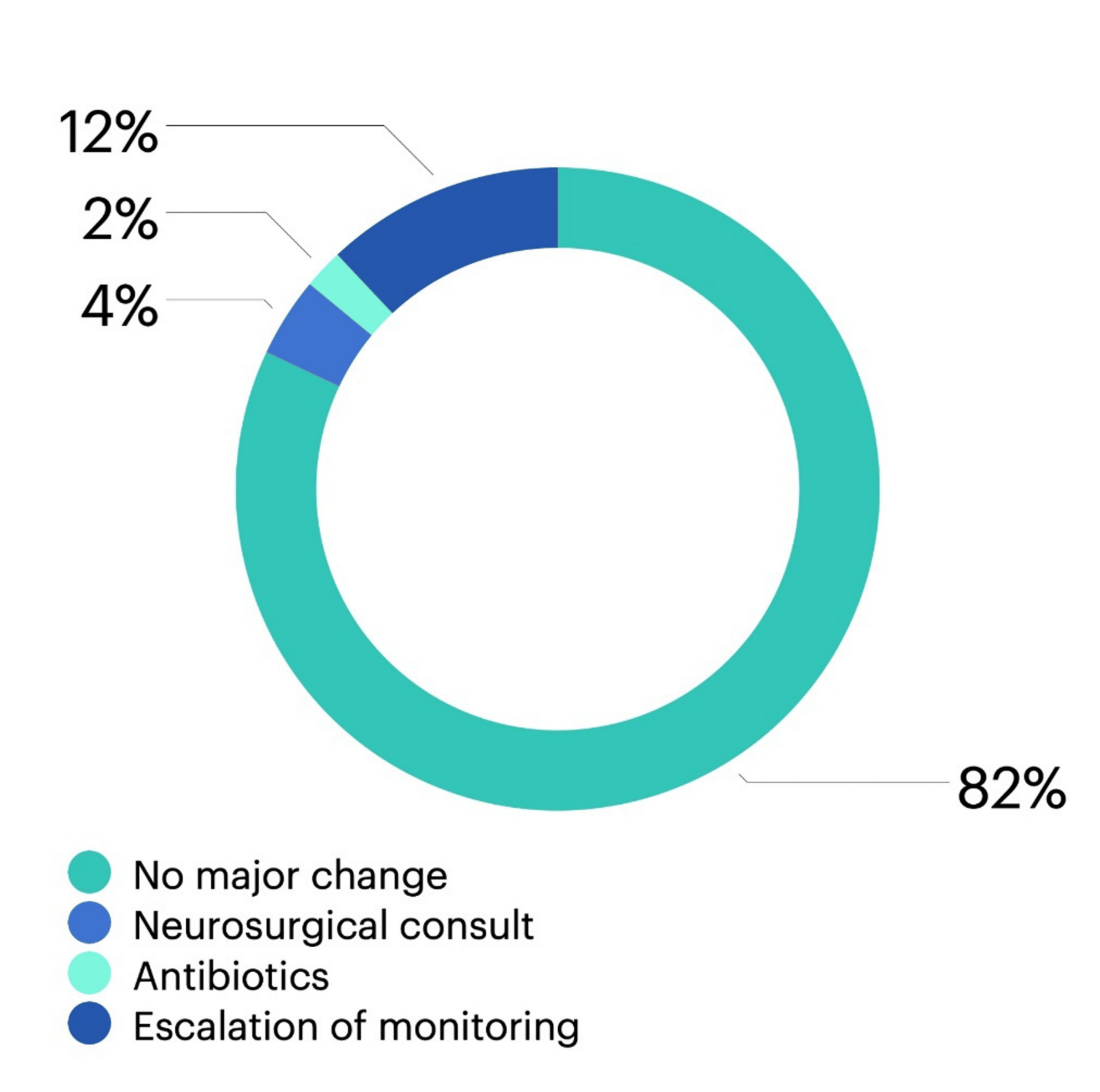
Distribution of management outcomes following neurosonography, demonstrating the proportion of scans that resulted in no major change versus those leading to alteration in clinical management.

Management impact was further assessed in the form of no major change, neurosurgical consult, antibiotics, and escalation of monitoring, as shown in Figure 12. On the follow-up of these patients, we discovered that 24 (18%) scans directly influenced management, which had significant clinical implications [14,15]. Neurosurgical consultation was triggered by cases of high-grade IVH and progressive hydrocephalus; antibiotic initiation or escalation was driven by evidence of meningitis or ventriculitis; and intensified monitoring was established in neonates with evolving haemorrhagic or white matter changes.

These findings reinforce the position that neurosonography can function not merely as a diagnostic test but as a clinical decision-support tool whose impact extends well beyond the imaging report itself. To maximise this clinical value, we propose that each neurosonography report should incorporate three core components: a clearly stated clinical indication, a graded severity or classification of any pathological finding, and explicit, disease-specific action recommendations or outcome-oriented intervention triggers. However, this is an exploratory framework pending prospective validation.

Underutilisation analysis

**Figure 13:**
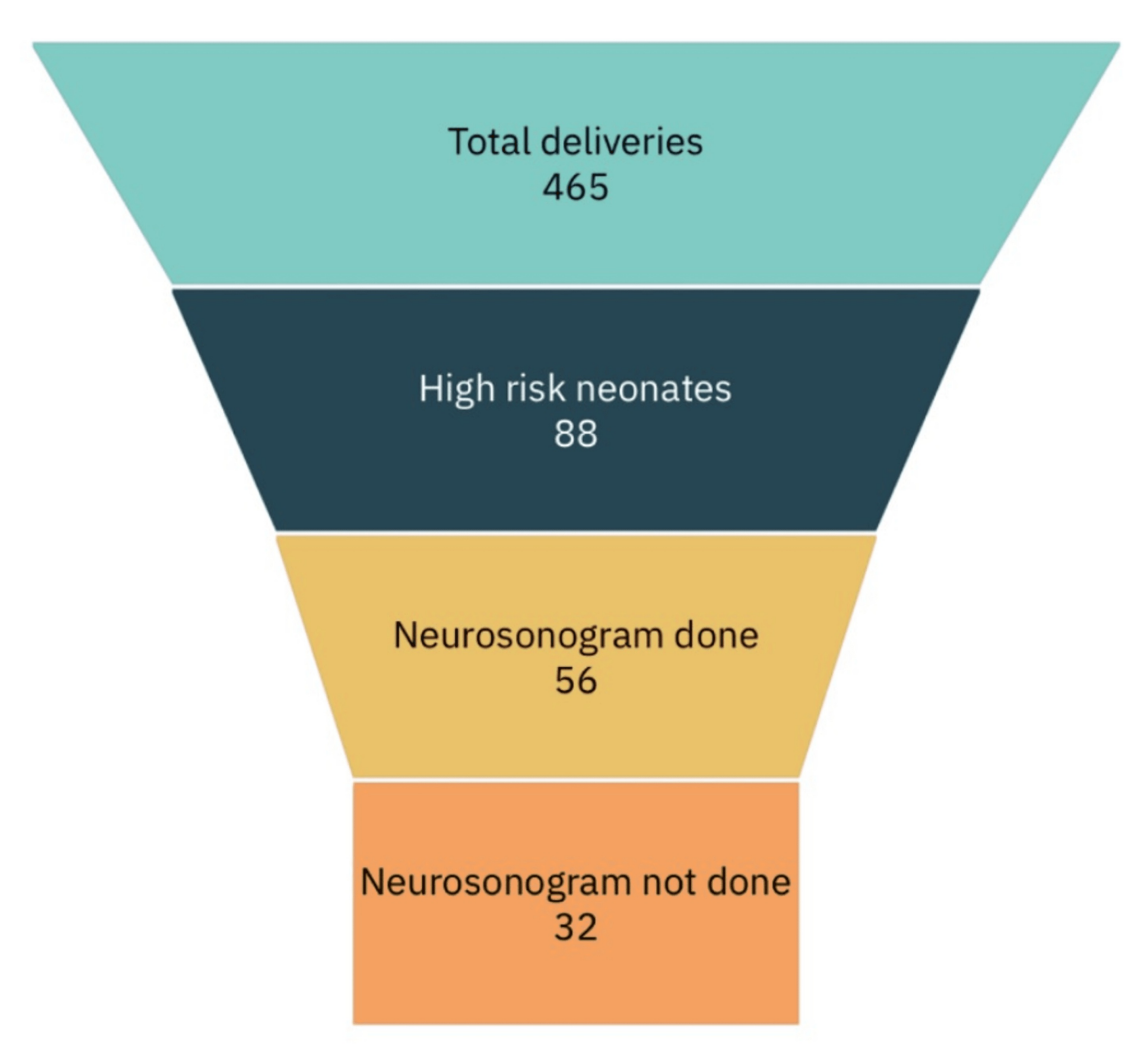
Funnel diagram illustrating the underutilisation of neurosonography among high-risk classified neonates.

To evaluate the extent of underutilisation, all in-house deliveries during the same 12-month study period were reviewed and classified according to neurosonography indications derived from the AIUM and AAP criteria [2,3]. Risk criteria included prematurity at or below 32 weeks of gestational age, very low birth weight, perinatal asphyxia, suspected sepsis or meningitis, and congenital anomalies. Of the 465 total deliveries in this period, 88 neonates met high-risk criteria and should therefore have undergone neurosonography. Of these, 56 received the scan, while 32 (36%) did not, as shown in the funnel diagram (Figure 13). This represents a substantial proportion of missed high-risk neonates and underscores the real-world gap between guideline recommendations and clinical practice. Delayed or absent neuroimaging in this group carries meaningful consequences, as conditions such as GMH, PVL, and early hydrocephalus are time-sensitive diagnoses where earlier detection may facilitate earlier intervention, and existing literature suggests this is associated with improved neurodevelopmental outcomes [16,17]. However, our study design cannot directly establish this causal relationship.

## Discussion

Our audit reveals considerable variation in neurosonography utilisation, reflecting imaging decisions made without a standardised framework. While two-thirds of referrals met clear guideline-backed criteria, approximately one-quarter were either possibly appropriate or inappropriate, indicating both overuse in low-yield scenarios and missed opportunities in neonates who warranted imaging. Diagnostic yield was significantly higher in appropriately referred neonates (51% vs. 18%, p = 0.038), suggesting an association between referral quality and imaging value in this cohort. However, this observational finding should be interpreted cautiously, given the single-centre design and modest subgroup sizes.

The underutilisation finding is particularly concerning. Of 88 neonates meeting high-risk criteria during the study period, 32 (36%) did not receive neurosonography. In conditions such as germinal matrix haemorrhage and early white matter injury, the window for effective intervention is narrow. Missed scans in this group represent missed opportunities for timely neurosurgical consultation, adjustment of medical management, and enrolment in neurodevelopmental follow-up. This gap between guideline recommendations and clinical practice is the most modifiable finding of our audit. Addressing it does not require new infrastructure, only a structured referral trigger at the point of clinical decision-making.

The abnormalities identified in our cohort, including GMH-IVH, PVL, hydrocephalus, and suspected intracranial infection, carried important clinical implications. Eighteen percent of examinations directly influenced patient management through neurosurgical referral, targeted medical therapy, or enrolment in neurodevelopmental follow-up programmes. These findings support the role of neurosonography as a clinically relevant imaging modality when appropriately indicated.

**Figure 14:**
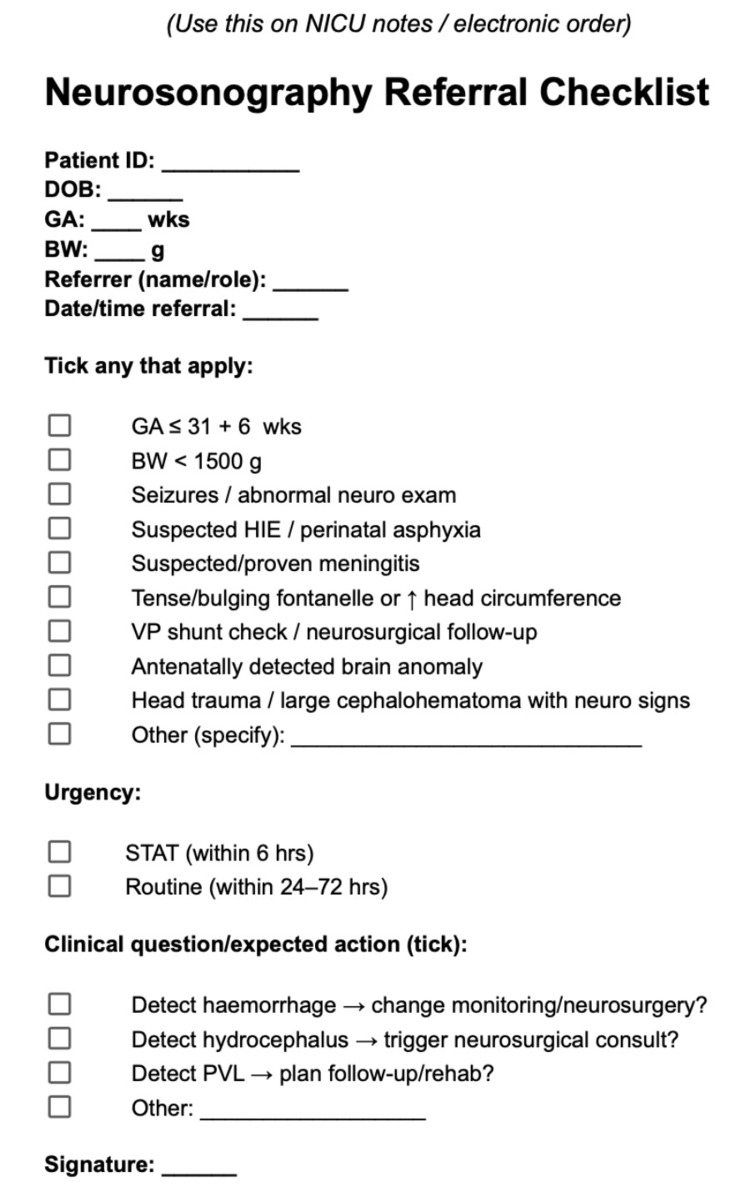
Proposed neurosonography referral checklist for optimal use of neurosonography in the neonatal intensive care unit (NICU)/neonatal wards, preventing its underutilisation.

**Figure 15:**
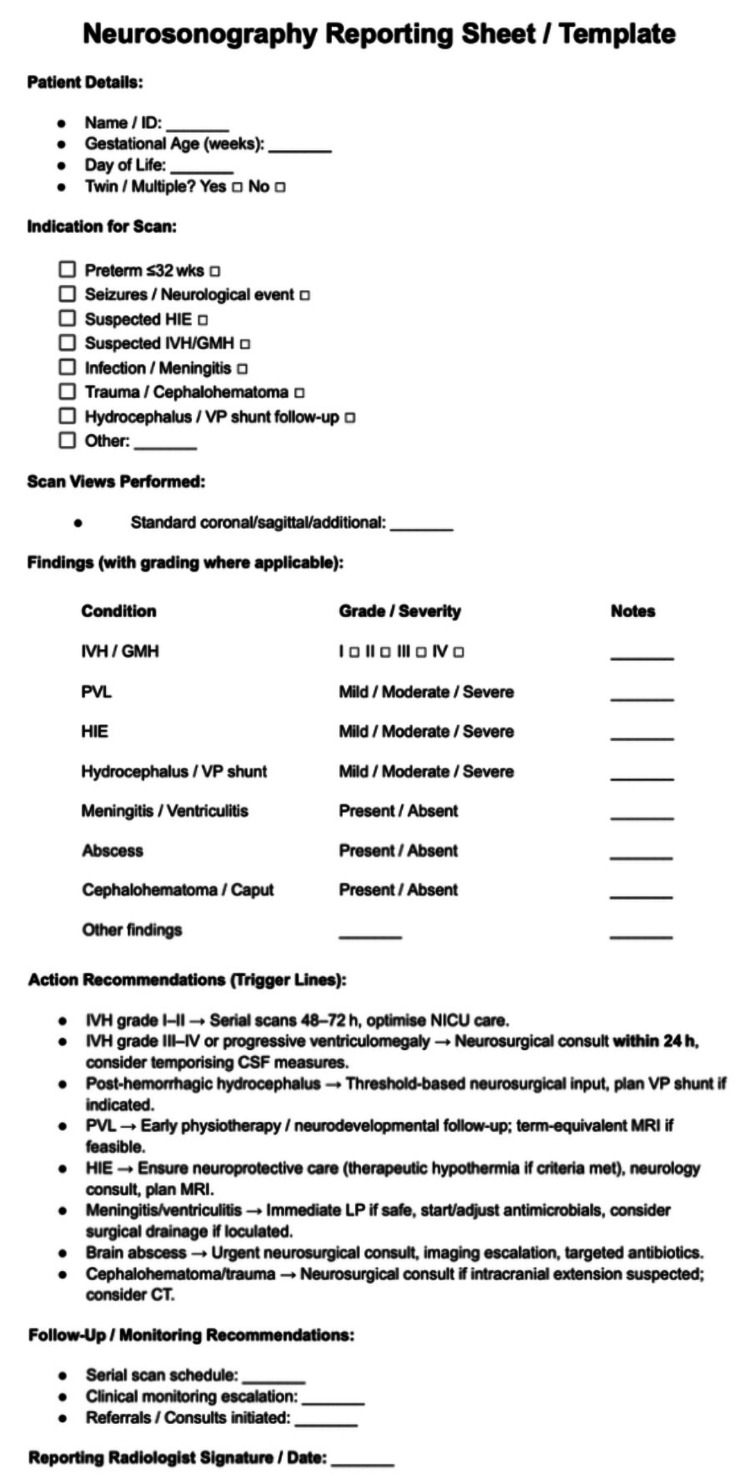
Neurosonography reporting template with disease-specific action triggers. It includes indication, grade/severity, and clear action recommendations.

Based on these findings, we developed a structured referral checklist (Figure 14) incorporating evidence-based criteria from international neonatal neurosonography guidelines [2-5]. The checklist is designed to be used at the point of clinical decision-making, prompting the clinician to verify the indication, categorise urgency, and pre-specify the expected management consequence before ordering the scan. On the reporting side, we also propose embedding disease-specific action trigger lines within a structured reporting template (Figure 15). Each report should state the indication, the grade or severity of any finding, and a clear recommended action. This creates a direct link between the imaging result and its clinical response. It must be emphasised that both tools are proposed as exploratory instruments derived from a single-centre retrospective audit. They have not undergone prospective validation and should not be adopted for broader implementation without multicentre evaluation of their feasibility, reliability, and clinical impact. 

To the best of our knowledge, there is limited published literature defining a guideline-backed referral protocol combined with an action-embedded reporting template of this kind. The current AIUM and AAP guidelines [2,3] provide broad indications for scanning but do not specify the referral pathway, urgency grading, or reporting format needed to translate those indications into consistent clinical practice. Our protocol is an attempt to bridge that gap.

Our study has several limitations worth acknowledging. It is a single-centre retrospective audit, and our centre is not a dedicated paediatric neurology unit. We have a relatively modest volume of in-house deliveries and NICU admissions compared to larger teaching hospitals. The small numbers in some subgroups limit the statistical power of certain comparisons. Data were collected from pre-existing clinical records, carrying an inherent risk of incomplete documentation. Inter-centre variability in referral culture and scan technique could not be assessed. The findings should therefore be regarded as hypothesis-generating and observational rather than confirmatory. Prospective multicentre validation of the proposed tools would be a valuable next step. As part of an ongoing quality-improvement initiative, a repeat audit is planned after one year of implementation of the proposed referral and reporting framework to evaluate its impact on referral practices and diagnostic yield.

## Conclusions

We conclude that neurosonography can prove to be an essential tool for NICU care. Its clinical impact may improve when guided by structured referral protocols and standardised action-oriented reporting. This audit illustrates that such structured approaches could strengthen neurosonography's role as an actionable tool in neonatal care. 
